# Persimmon leaf extract alleviates chronic social defeat stress-induced depressive-like behaviors by preventing dendritic spine loss via inhibition of serotonin reuptake in mice

**DOI:** 10.1186/s13020-022-00609-4

**Published:** 2022-06-06

**Authors:** Hui Yu, Shumin Shao, Junnan Xu, Haibiao Guo, Zhangfeng Zhong, Jiangping Xu

**Affiliations:** 1 Guangdong Provincial Key Laboratory of New Drug Screening, School of Pharmaceutical Sciences, Southern Medical University, Guangzhou, 510515 China; 2 Macau Centre for Research and Development in Chinese Medicine, Institute of Chinese Medical Sciences, University of Macau, Macao, 999078 SAR China; 3 Department of Neurobiology, School of Basic Medical Sciences, Southern Medical University, Guangzhou, 510515 China; 4 Hutchison Whampoa Guangzhou Baiyunshan Chinese Medicine Co., Ltd, Guangzhou, 510515 China

**Keywords:** Persimmon leaf, Depression, Serotonin reuptake, Dendritic spines, cAMP/CREB/BDNF signaling pathway, Microbiota–gut–brain axis

## Abstract

**Background:**

Fresh or dried Persimmon leaves (*Diospyros kaki *Thunb*.*) exhibit preventive effects on cardiovascular and cerebrovascular diseases. However, their antidepressant effects and underlying mechanisms are unclear. Thus, we investigated mechanisms responsible for Persimmon leaf extract (PLE) activity on chronic social defeat stress (CSDS)-induced depressive-like behaviors in mice.

**Methods:**

CSDS was used as a mouse model of depression. We performed the sucrose preference test (SPT), forced swim test (FST), and tail suspension test (TST) to identify depressive-like behavior. Spine density and dendritic morphology were assessed using Golgi staining. Neurochemicals were quantified by microdialysis, doublecortin by immunofluorescence, and cAMP using an ELISA kit. Finally, the levels of cortical proteins of phosphorylated cAMP-response element binding protein (CREB), brain-derived neurotrophic factor (BDNF), postsynaptic density synapsin-1 and protein 95 (PSD95) were quantified by western blot. 16S rRNA gene sequencing was used to detect fecal microbiota.

**Results:**

Treatment of CSDS-subjected mice with PLE (30.0–60.0 mg/kg, *i.g.*) enhanced sucrose preference, decreased immobility times in the TST and FST but did not affect locomotor activity. Furthermore, persistent social defeat stress decreased dendritic spine density and dendritic length in the brain, as well as decreased PSD95 and synapsin-1 expression. PLE, interestingly, inhibited dendritic spine loss and increased synaptic protein levels. PLE also increased brain levels of 5-HT, cAMP, phosphorylated (p)-CREB, BDNF, PSD95, and synapsin-1 in mice subjected to CSDS. Furthermore, PLE increased their doublecortin-positive cell count in the hippocampal dentate gyrus. CSDS mice represented a distinct fecal microbiota cluster which differed compared with normal C57BL/6J mice, and the phenotype was rescued by PLE.

**Conclusions:**

PLE alleviated CSDS-induced depressive behaviors and spinal damage by suppressing serotonin reuptake and activating the cAMP/CREB/BDNF signaling pathway. Simultaneously, PLE influenced the composition of the fecal microbiota in CSDS-subjected mice.

**Supplementary information:**

The online version contains supplementary material available at 10.1186/s13020-022-00609-4.

## Background

Depression is a chronic and recurrent affective and mental illness that has become the third most common disease in the world [1, 2]. Significant and persistent emotional downturns are its principal clinical features, which are often accompanied by anxiety, suppressed thoughts, delusions, or hallucinations, decreased attention and memory, and sleep disturbances [3]. The rapid pace of life and the increasing pressure of work make depression a common disease that seriously threatens human health [4, 5]. Despite continuous research, treating depression remains a challenge due to its unclear etiology and pathology, and antidepressants remain insufficient for broad therapeutic success [6–8]. Selected serotonin reuptake inhibitors, serotonin–norepinephrine reuptake inhibitors, and other chemical drugs are successful in about 50–70% of patients with serious depressive disorders [9–11]. However, these drugs vary in efficacy for different subtypes of depression and have drawbacks such as delayed onset, side effects, low efficiency, dependence, high rate of recurrence, and high price [12, 13]. Therefore, researchers seeking safer, less toxic, more effective, and cheaper antidepressants increasingly turn their attention to abundant and readily available, natural plants.

Persimmon (*Diospyros kaki Thunb.*) is a plant of the genus Diospyros of the Ebenaceae family [14]. Persimmon leaves contain a variety of active ingredients and nutrients, such as flavonoids [15], organic acids [16], coumarins, and triterpenes, which have potent preventive effects on cardiovascular and cerebrovascular diseases [17]. There is evidence that persimmon leaves can treat nervous system disorders, such as ischemic stroke [18] and Alzheimer’s disease [19], by regulating the immune function to inhibit inflammation and enhance neuroprotection. Persimmon leaf has been shown to protect myocardial cells, decrease inflammation, and reduce oxidative stress in metabolic disorders [20, 21]. Furthermore, a recent study showed that persimmon leaves could regulate platelet serotonin (5-HT) levels [22]. However, nothing is known regarding the activity of persimmon leaves on depression-related neurotransmitters and 5-HT release.

The 5-HT receptors 1A (5-HT1A), 1B (5-HT1B), and 7 (5-HT7) play a crucial role in the pathophysiology of depression [23, 24]. Furthermore, activation of postsynaptic 5-HT1A and 5-HT1B upregulates various signaling molecules such as cyclic adenosine monophosphate (cAMP), cyclic-AMP dependent protein kinase A (PKA), cAMP response element-binding protein (CREB), and brain-derived neurotrophic factor (BDNF) [25–28]. The cAMP/PKA/CREB signaling pathway controls various biological activities related to cAMP, and in particular emotion [29]. Postmortem experiments revealed that individuals with significant depression had low levels of phosphorylated CREB in both the hippocampus and the prefrontal cortex, but persistent antidepressant therapy restored the level of phosphorylated CREB [30]. In an acquired helplessness animal model, elevated CREB expression in the hippocampus dentate gyrus caused antidepressant-like effects in the forced swimming test (FST) [31, 32]. Activated CREB increases the production of BDNF, a powerful trophic factor that regulates synaptic plasticity and preserves the shape of neurons [33]. Antidepressants may increase or normalize the otherwise low levels of cerebral BDNF in patients with major depressive disorder [27, 34, 35]. In neurons of newborn mice, cAMP/CREB/BDNF signaling is crucial in mediating neuroplasticity and contributes to antidepressant-like effects [36]. Thus, the present study aimed to elucidate the antidepressant activity of persimmon leaves in a chronic social defeat stress (CSDS) mouse model of depression to explore the underlying mechanisms.

## Methods and materials

### Animals

We obtained single-housed male CD-1 mice (4-month-old sexually experienced retired breeders) from Charles River Laboratories and adult male C57BL/6 mice (22–25 g) from the Laboratory Animal Center of Southern Medical University (Guangzhou, China). The experimental protocols minimized the number of animals and their suffering. The mice were kept in a room with a 12-h light/dark cycle, at 22–23 °C and 55–65% humidity. Mice had free access to food (normal raw chow) and water for 1 week before the trials. All experimental protocols strictly followed the NIH Guidelines for the Care and Use of Laboratory Animals and were approved by the Animal Care and Use Ethics Committee of the Southern Medical University.

### Reagents and treatments

**Fig. 1:**
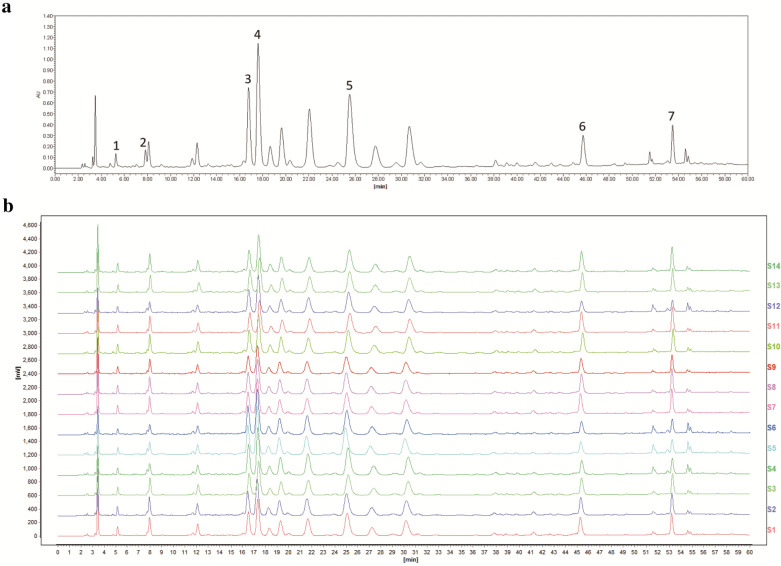
High-performance liquid chromatography chromatograms and fingerprint of Persimmon leaf extract (PLE) and establishment of relative peaks. **a** HPLC chromatograms of PLE, 1-protocatechuic acid, 2-furoic acid, 3-isoquercitrin, 4-hyperin, 5-astragalin, 6-quercetin, and 7-kaempfetol. **b** Fingerprint of PLE

Persimmon leaf extract (PLE) was supplied from Hutchison Whampoa Guangzhou Baiyunshan Chinese Medicine Co., Ltd. (#C21P005, Guangzhou, China); Fig. 1 shows the HPLC chromatograms and the fingerprint of PLE. Fluoxetine was obtained from Aladdin (#F189157, Shanghai, China). PLE and fluoxetine were diluted in vehicle (0.5% DMSO, 0.5% carboxymethylcellulose sodium) to obtain working solutions. The drugs were prepared freshly before use. The other chemicals used were of analytical grade.

We obtained the GolgiStain™ Kit from FD NeuroTechnologies, Inc., (#PK401, Columbia, USA), ELISA Kits from Cusabio Biotech Co., Ltd. (#CSB-E08300m, Wuhan, China), and a BCA protein assay kit from Thermo Fisher Scientific (#23,235, Waltham, MA). A protease inhibitor cocktail (#P8849), and the anti-BDNF (#SAB2108004), and anti-β-actin (#39199) antibodies were obtained from Sigma-Aldrich (St. Louis, MO). We purchased a BCA protein assay kit (#23235) from Thermo Fisher Scientific (Waltham, MA), and the anti-phospho-CREB (#9198) and anti-CREB (#9197) antibodies from Cell Signaling Technology, Danvers, CT). Anti-PSD95 (#ab18258) and anti-synapsin1 (#ab64581) antibodies were obtained from Abcam (Cambridge, UK). Finally, the OCT compound (#4583) was purchased from Tissue-Tek (Torrance, UK), guinea pig anti-doublecortin (#ab2253) from Millipore (Massachusetts, USA), and Alexa Fluor 488 AffiniPure goat anti-guinea pig IgG (H+L) (#100-545-003) were obtained from Jackson (Pennsylvania, USA).

### Experimental groups and social defeat stress animal model

**Fig. 2:**
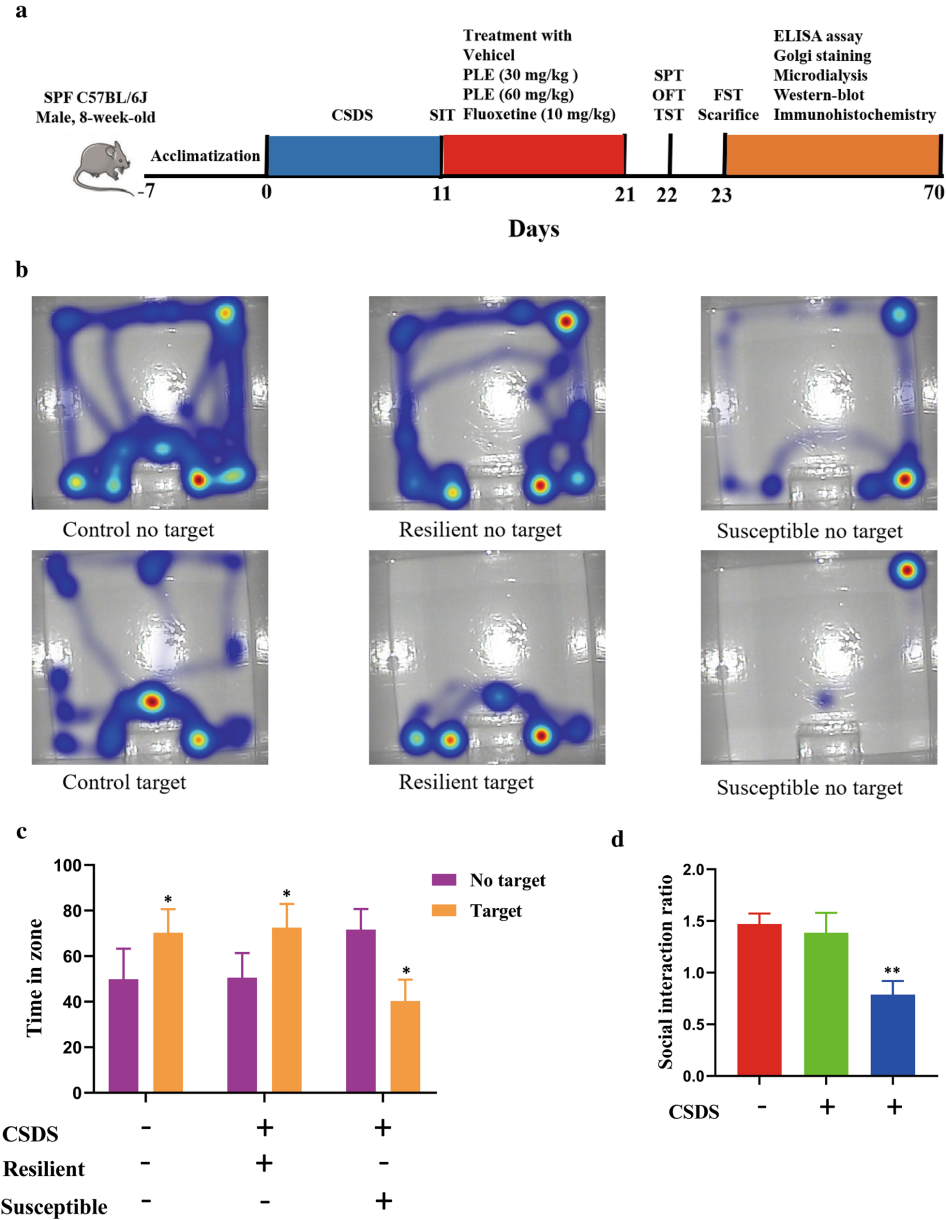
Experimental procedures and social interaction test. **a** Schematic timeline of the experimental procedure. **b** Time spent in the interaction zone. The data are presented as mean ± SD. *P < 0.05 vs. No target. **c** Social interaction ratio. Data are presented as mean ± SD. **P < 0.01 vs. Control group

CSDS was used as a murine model of depression. Figure 2a illustrates the experimental design. After 1 week of acclimatization, the mice underwent 10 days of the CSDS procedure, then were housed in isolation for 24 h and subjected to the social interaction test (SIT) on day 11. Next, we selected susceptible mice for follow-up experiments (Fig. 2b, c). We randomly assigned 40 susceptible mice to four groups (n = 10 each): (1) CSDS + vehicle; (2) CSDS + L-PLE (low-dose PLE (30.0 mg/kg)); (3) CSDS + H-PLE (high-dose PLE (60.0 mg/kg)); and (4) CSDS + fluoxetine (10.0 mg/kg). Mice received PLE or fluoxetine (*i.g.*) once a day for 10 consecutive days. Mice in the Vehicle (Control group) and CSDS + vehicle groups received the same volume of vehicle. After the procedure, we performed the sucrose preference test (SPT), open field test (OFT), and tail suspension test (TST) on day 21, and the FST on day 22. Next, we sacrificed the animals for biochemical analysis and Golgi staining. All experiments were performed in a blinded manner.

### CSDS procedure

We performed the CSDS procedure as previously described [37]. C57BL/6 mice were used as “intruder” animals and were exposed to CD-1 for 10 days. The defeated mice were then each subjected to ongoing psychological stress from a CD-1 mouse in a shared home cage for the following 24 h using a transparent perforated barrier that allowed for visual, olfactory, and auditory interaction.

### Social interaction test

We identified sensitive mice using the social interaction test (SIT). Mice were placed in a unique, open-field arena with an interaction zone for two 150-s trials. An empty cage was placed in the interaction zone in the first ‘no target’ trial. Next, a new CD-1 mouse was placed inside the cage in the interaction zone for a ‘target trial’. Ethovision video tracking software was used to track the time spent in the interaction zone (Noldus Technology). We calculated the social interaction ratio by dividing the time spent in the interaction zone in the ‘target trial’ by that of the ‘no target’ trial. Susceptible mice were defined as having a social interaction ratio < 1.

### Sucrose preference test

We performed the SPT as previously described with a minor modification [38]. On the first day, each cage was filled with two bottles of 1% sucrose solution. Mice were given 24 h to adapt to this sugar solution. On the second day, one of the bottles was replaced with pure water, and the positions of the two bottles were changed during this time (24 h). Mice received no water or food for 24 h and were then tested for 12 h. We calculated sucrose preference as follows: (weight of sucrose solution ingested)/(weight of water ingested + weight of sucrose solution ingested)/(weight of sucrose solution ingested)/(weight of water ingested + weight of sucrose solution ingested)/(weight of water ingested + weight of sucrose solution ingested).

### Open field test

We analyzed the effects of PLE on the spontaneous locomotor activity of mice through an OFT. The mice were placed in a white wooden box (40 × 30 × 20 cm) for 5 min. Using an open-field experimental video analysis system (Smart 3.0, Video tracking system, Panlab, Barcelona, Spain), we recorded the rear number (the number of vertical rearing movements within 5 min) and the crossing number (the number of times the animal passed from one square to another within 5 min) for each animal.

### Tail suspension test and forced swim test

The TST and FST are widely used approaches to assess depressive behavior in animals [39]. For the TST, mice were suspended 20 cm above the floor using adhesive tape positioned 3 cm from the tip of their tails, and the total time of immobility during the 6 min session was recorded. For the FST, mice were placed in a transparent glass cylinder (10 cm in diameter, 25 cm in height) filled with fresh water at 23 °C, and the total time of immobility during the last 4 min of the 6 min session was recorded. A camera with a video analysis system was used to capture immobility time in both tests.

### Microdialysis studies

We implanted concentrated dialysis probes in the ventromedial prefrontal cortex of mice. Microdialysis tests were performed on freely moving animals 24 h after implantation. Six 20-min fractions were obtained after a 180-min stabilization period to achieve basal values, and another six samples were collected after the *i.g.* administration of treatments. 5-HT, norepinephrine (NE), and gamma-aminobutyric acid (GABA) were quantified using high-performance liquid chromatography (HPLC). After the tests, mice were euthanized and brain tissue was processed using standard histological procedures (cresyl violet staining) to ensure the proper placement of the dialysis probe placement. Mice with incorrect probe placement were discarded (< 10%).

### Golgi staining and dendrite analysis

We performed Golgi staining using an FD Rapid Golgistain Kit (PK401A, FD NeuroTechnologies, Columbia, MD) following the manufacturer’s instructions. The brains of the mice were removed as quickly as possible from the skulls and were rinsed in double-distilled water to remove blood from the surface. The brains were then soaked in equal volumes of solutions A and B and kept at room temperature for 5 weeks in the dark. The brains were then transferred to solution C and kept in the dark for 7 days at room temperature. The brains were then sectioned into 100 μm slices using a freezing microtome and mounted on gelatin-coated glass coverslips with solution C. The slices were incubated in a combination solution (solution D:solution E:distilled water, 1:1:2) for 10 min after drying at room temperature. Subsequently, the slices were dehydrated with 50%, 75%, 95%, and 100% ethanol.

Image J was used to trace 10 neurons in the cortex for quantitative analysis. Pyramidal neurons in the cortex were photographed under intense field illumination with a 100× oil immersion objective for dendritic spine analysis. A laser confocal microscope (LSM880 with Airyscan; Carl Zeiss, Oberkochen, Germany) coupled to a computer running the ZEN program was used to trace neurons. The neurons chosen for the tracing had a fully stained and isolated cell body and displayed fully stained and complete dendritic arbors. Dendritic traces were quantified using ImageJ software. The Neuron J plugin for Image J was used to count dendritic spines. The spines were counted at a magnification of 1000. The total number of spines along a 50 μm dendritic length was used to calculate spine density. The density of the spine was studied using pyramidal neurons from the cortex. The spines of the apical dendrites were counted. We used a common two-dimensional method to assess spine density, which allowed us to directly compare the treatment groups examined in the same way. The Sholl analysis plugin for Image J was used to determine the overall dendritic length and the number of branching points. Under 400×, the Sholl traced neurons and created a series of concentric rings around the neuron bodies. The beginning radius was set at 10.00 μm, with a radius interval of 10 μm between circles. The dendrites intersecting the concentric rings were counted.

### Enzyme-linked immunosorbent assay

Cortical cAMP was quantified using an ELISA kit according to the manufacturer’s instructions. The samples were tested in duplicate and the cAMP levels were adjusted to total protein.

### Western blot

Western blot analysis was performed as previously described, with minor changes. The mice’s cortex was homogenized in RIPA lysis buffer (containing 1% protease inhibitor cocktail and 1% phosphatase inhibitor cocktail) and centrifuged at 12,000×*g* for 10 min. A BCA protein assay kit was used to determine the total protein content. We then separated the proteins by electrophoresis on a sodium dodecyl sulfate-polyacrylamide gel (SDS-PAGE) and transferred the protein bands onto polyvinylidene fluoride membranes. The membranes were incubated for 2 h at room temperature in phosphate buffered solution with Tween 20 (TBST) containing 5% skim milk to inhibit nonspecific binding sites. The membranes were then washed three times with PBST before being incubated overnight at 4 °C with the appropriate primary antibodies, such as anti-phospho-CREB (1:1000), anti-CREB (1:1000), anti-BDNF (1:1000), anti-PSD95 (1:1000), anti-synapsin-1 (1:1000), or anti-β-actin (1:1000). The membranes were then rinsed with TBST and incubated with appropriate secondary antibodies for 1 h at room temperature. Finally, the bands were visualized using a Kodak Digital Science ID (Kodak, Rochester, NY) and quantified with Image J software (National Institutes of Health, Bethesda, MD).

### Immunofluorescence

Mice were perfused with 4% paraformaldehyde (PFA) and ice-cold phosphate buffered saline (PBS). The brains were post-fixed in 4% PFA and dehydrated in graded sucrose solutions. The tissue was embedded in OCT compounds and cut into 40 μm-brain slices using a frozen slicer (Leica, Germany, #CM1850-1-1). Sections were washed with PBS and then soaked in 0.3% Triton for 15 min. After blocking for 2 h at room temperature with 5% bovine serum albumin, sections were incubated overnight at 4 °C with guinea pig anti-doublecortin. The sections were then incubated for 2 h at room temperature with a suitable secondary antibody (Alexa Fluor 488 AffiniPure goat anti-guinea pig IgG (H + L) or Alexa Fluor 488 AffiniPure goat anti-guinea pig IgG (H + L)) and 10 min with DAPI. The images were analyzed with a confocal microscope (Nikon, Japan). Nikon Imaging Elements software was used to examine six fields from each group for immunofluorescence quantification. Doublecortin positive cells (DCX^+^) were counted in each field.

### 16S rRNA gene sequencing

Before the experiment, feces samples were collected from the experimental mice and kept at – 80 °C. Genomic DNA was extracted from the samples using the MagPure Soil DNA KF Kit (Cat# D6356-F-96-SH, Qiagen, Venlo, The Netherlands). Using agarose gel electrophoresis and NanoDrop 2000 spectrophotometer (Thermo Fisher Scientific, Waltham, MA, USA), the purity and concentration of the isolated DNA were determined. Primers targeting V3–V4 regions (5′-TACGGRAGGCAGCAG-3′, 5′-GGGTATCTAATCCT-3′) were used to amplify bacterial 16S rRNA gene, the reverse primer contained a sample barcode and both primers were connected with an Illumina sequencing adapter. The DNA were then Sequenced on an Illumina NovaSeq6000 with two paired-end read cycles of 250 bases each. (Illumina Inc., San Diego, CA; OE Biotech Company; Shanghai, China). Raw sequencing data were in FASTQ format. The QIIME1 package (http://qiime.org/) was used to evaluate and selected representative reads of each OTU, and all representative sequences were annotated and blasted against Silva database Version 128 (https://www.arb-silva.de/). The microbial diversity in feces samples was estimated using the alpha diversity that include Observed-species index and Shannon index. The binary jaccard distance matrix performed by QIIME software was used for binary jaccard Principal coordinates analysis (PCoA). The 16S rRNA gene amplicon sequencing and analysis were conducted by OE Biotech Co., Ltd. (Shanghai, China).

### Statistical analysis

All data were analyzed using SPSS version 22.0 (SPSS Inc., Chicago, IL) and the data were presented as mean ± standard deviation (SD). Data were analyzed using one-way ANOVA followed by Bonferroni’s post hoc test. Differences were considered significant when P < 0.05.

## Results

### PLE alleviated depressive-like behaviors in mice exposed to CSDS

**Fig. 3:**
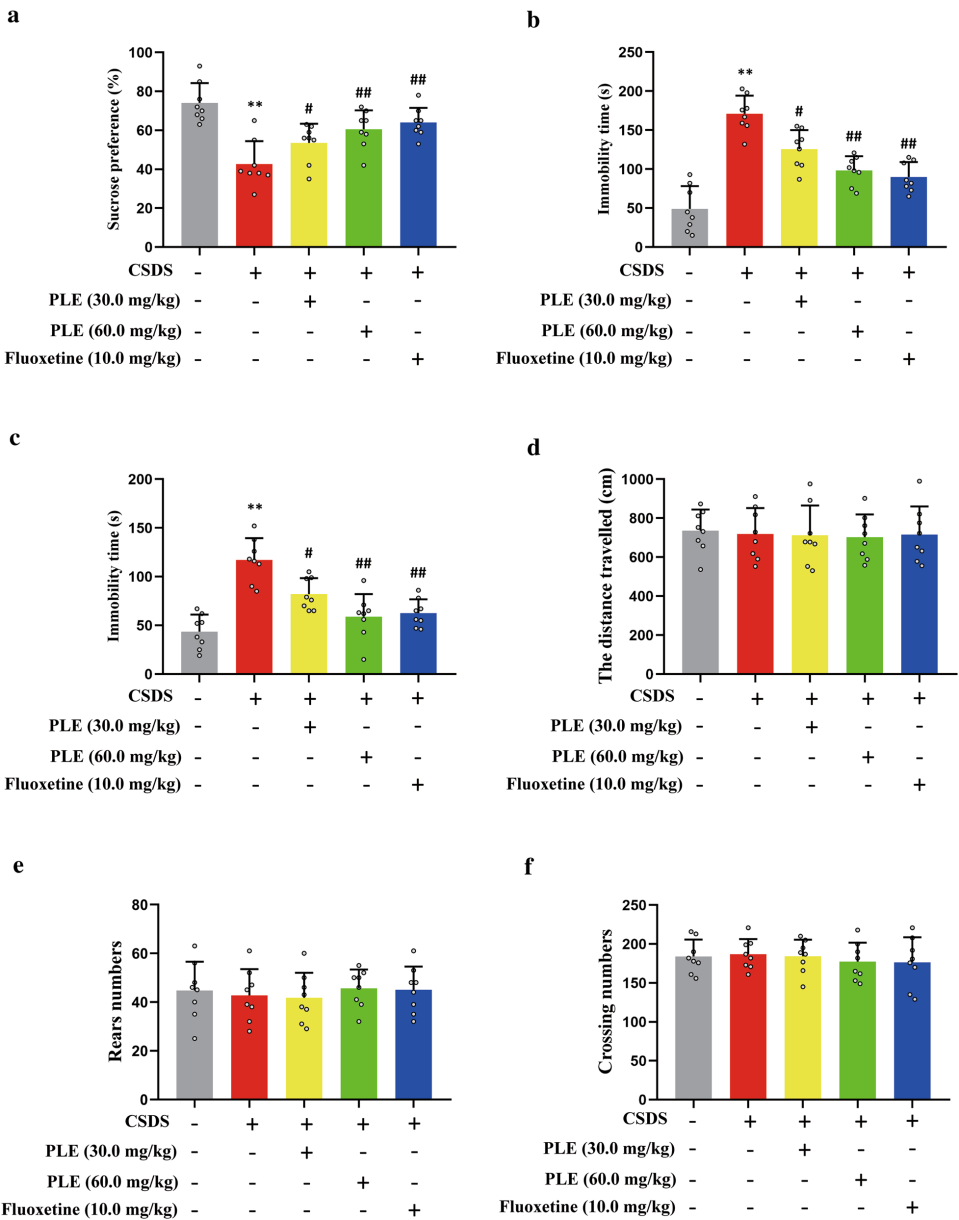
Antidepressant-like effect of Persimmon leaf extract (PLE) in mice subjected to chronic social defeat stress (CSDS). **a** Sucrose preference ratio in the sucrose preference test (SPT). **b** Immobility times in the tail suspension test (TST). **c** Immobility times in the forced swim test (FST). **d** Distance traveled in the open field test (OFT). **e** Rear numbers in the OFT. **f** Crossing numbers in the OFT. Data are presented as mean ± SD (n = 8 in each group). **P < 0.01 vs. Vehicle group; ^#^P < 0.05, ^##^P < 0.01 vs. CSDS + Vehicle group

We investigated the effects of PLE in mice with depressive-like behavior. Mice received PLE (30 and 60 mg/kg) or fluoxetine as control once a day for 10 days. To assess depressive-like behaviors, we performed SPT, OFT, TST, and FST. First, the mice treated with vehicle alone showed a strong preference for the sucrose solution, while the CSDS-challenged mice showed only a slight preference for the same solution (P < 0.01, Fig. 3a), which confirmed that these animals displayed depressive-like behaviors. PLE and fluoxetine restored sucrose preference in CSDS mice. Second, compared to the vehicle group, mice in the CSDS + vehicle group had a longer immobility time in the TST (P < 0.01; Fig. 3b) and FST (P < 0.01, Fig. 3c). However, PLE and fluoxetine significantly reduced the immobility time in the TST (P < 0.05 and P < 0.01, respectively) and FST (P < 0.05 and P < 0.01, respectively). Finally, we observed that for mice subjected to CSDS, PLE did not influence the distance traveled (P > 0.05, Fig. 3d). Put differently, treatment with PLE did not affect the ability of the mice to move in the OFT (P > 0.05, Fig. 3e, f), implying that PLE did not produce drowsiness in mice. Thus, PLE had a significant antidepressant-like effect on mice.

### PLE increased the dendritic complexity and spine density in CSDS-exposed mice

**Fig. 4:**
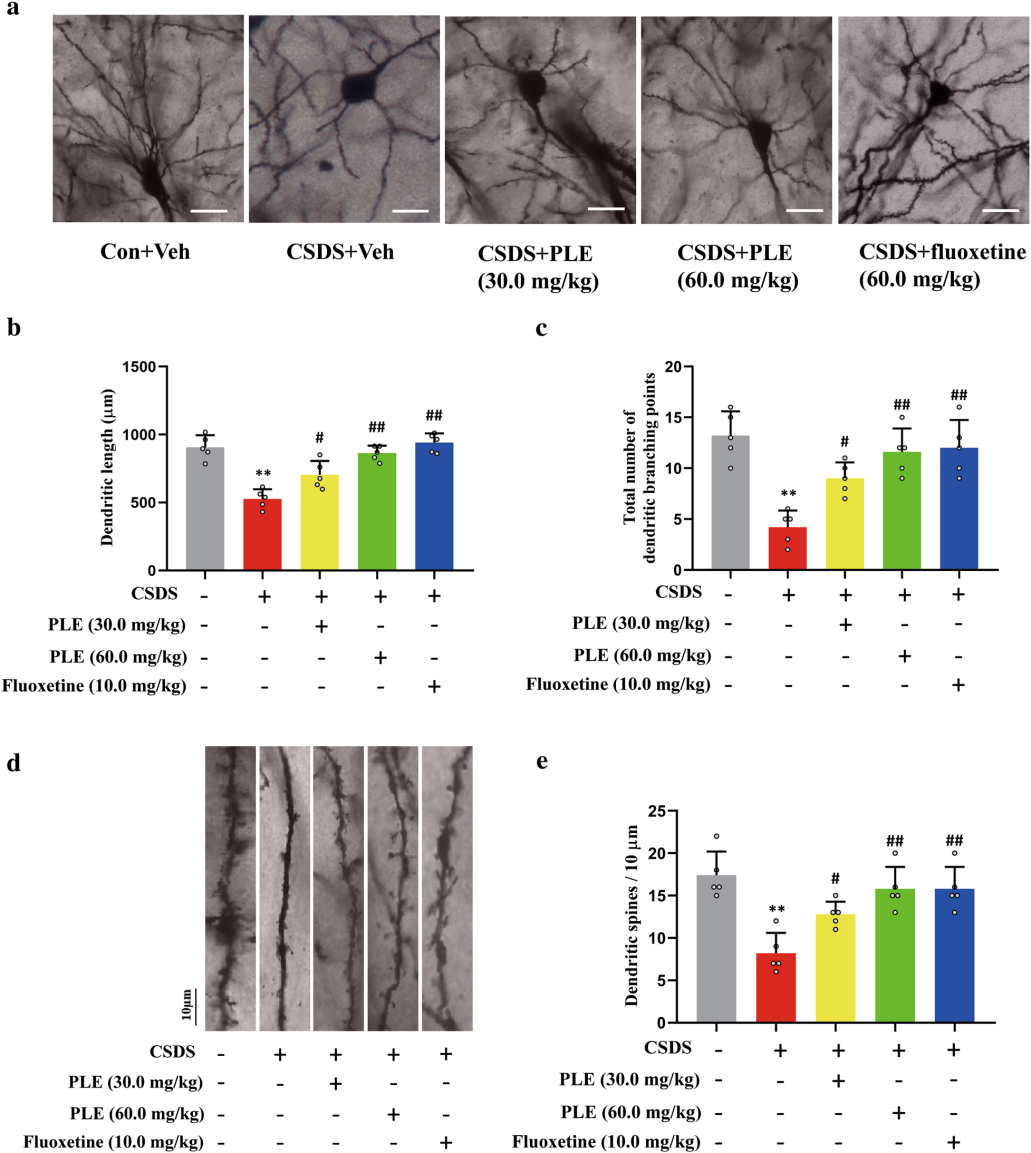
Effects of Persimmon leaf extract (PLE) on the synaptic plasticity in the cortex of mice subjected to chronic social defeat stress (CSDS). **a** Photomicrographs of Golgi-stained pyramidal neurons in the cortex of mice treated with PLE or fluoxetine for 10 days (scale bar: 50 μm). **b** Corresponding quantification data of the dendritic length in mice treated with PLE or fluoxetine for 10 days. **c** Total number of dendritic branching points in mice treated with PLE or fluoxetine for 10 days. **d** Photomicrographs of dendrite fragments with visible spines in the cortex of mice treated with PLE or fluoxetine for 10 days (scale bar: 10 μm). **e** Corresponding quantification data of dendritic spines. Data are presented as mean ± SD (n = 3 in each group). **P < 0.01 vs. Vehicle group; ^#^P < 0.05, ^##^P < 0.01 vs. CSDS + Vehicle group

Using Golgi staining, we assessed the complexity of the dendrite and spine density of cortical neurons in mice to study the influence of PLE on dendritic morphology. The mice in the Vehicle group had a long total dendritic length and abundant branching points, but the mice in the CSDS + vehicle group had significantly shorter total dendritic length (P < 0.01; Fig. 4a, b) and branching points (P < 0.01; Fig. 4c). In contrast, treatment with PLE and fluoxetine both significantly improved the overall dendritic length (P < 0.05, P < 0.01; Fig. 4a, b) and branching points (P < 0.05, P < 0.01; Fig. 4c). Additionally, we found that spine density varied between groups (Fig. 4d, e). The PLE-treated groups had a higher spine density compared to the CSDS + vehicle group. Thus, PLE effectively restored CSDS-induced dendritic complexity and reductions in spine density.

### PLE enhanced the PSD95 and synapsin-1 levels in CSDS-subjected mice

**Fig. 5:**
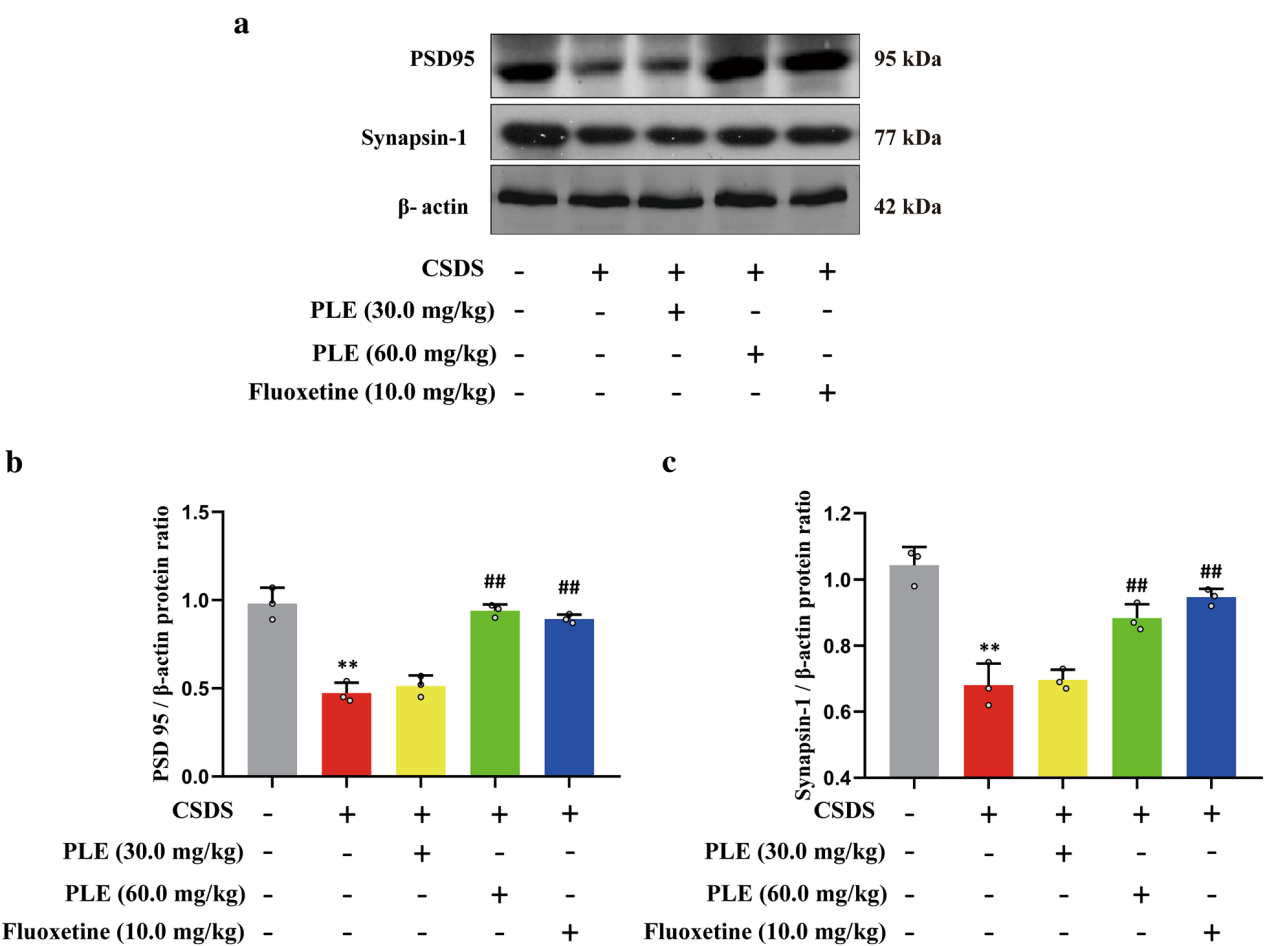
Effects of Persimmon leaf extract (PLE) on cortical levels of PSD95 and synapsin-1 protein of mice subjected to chronic social defeat stress (CSDS). **a** Examples of original western blot bands showing cortical PSD95 and synapsin-1 expression. **b** Relative levels of PSD95 protein. **c** Relative levels of synapsin-1 protein. Data are presented as mean ± SD (n = 3 in each group). **P < 0.01 vs. Vehicle group; ^#^P < 0.05, ^##^P < 0.01 vs. CSDS + Vehicle group

Next, we measured PSD95 and synapsin-1 protein levels in the cortex of animals subjected to CSDS to see how PLE affected the expression of synapse-related proteins. CSDS treatment induced a significant decrease in PSD95 (P < 0.01, Fig. 5a, b) and synapsin-1 (P < 0.01, Fig. 5a, c) protein levels compared to mice in the Vehicle group. Furthermore, 10 days of PLE or fluoxetine treatment resulted in a significant increase in the expression of PSD95 (P < 0.05, P < 0.01, Fig. 5a, b) and synapsin-1 (P < 0.05, P < 0.01, Fig. 5a, c). Thus, the antidepressant-like activity of PLE was associated with the synthesis of synaptic proteins such as PSD95 and synapsin-1.

### PLE inhibited 5-HT reuptake in the cortex of mice exposed to CSDS

**Fig. 6:**
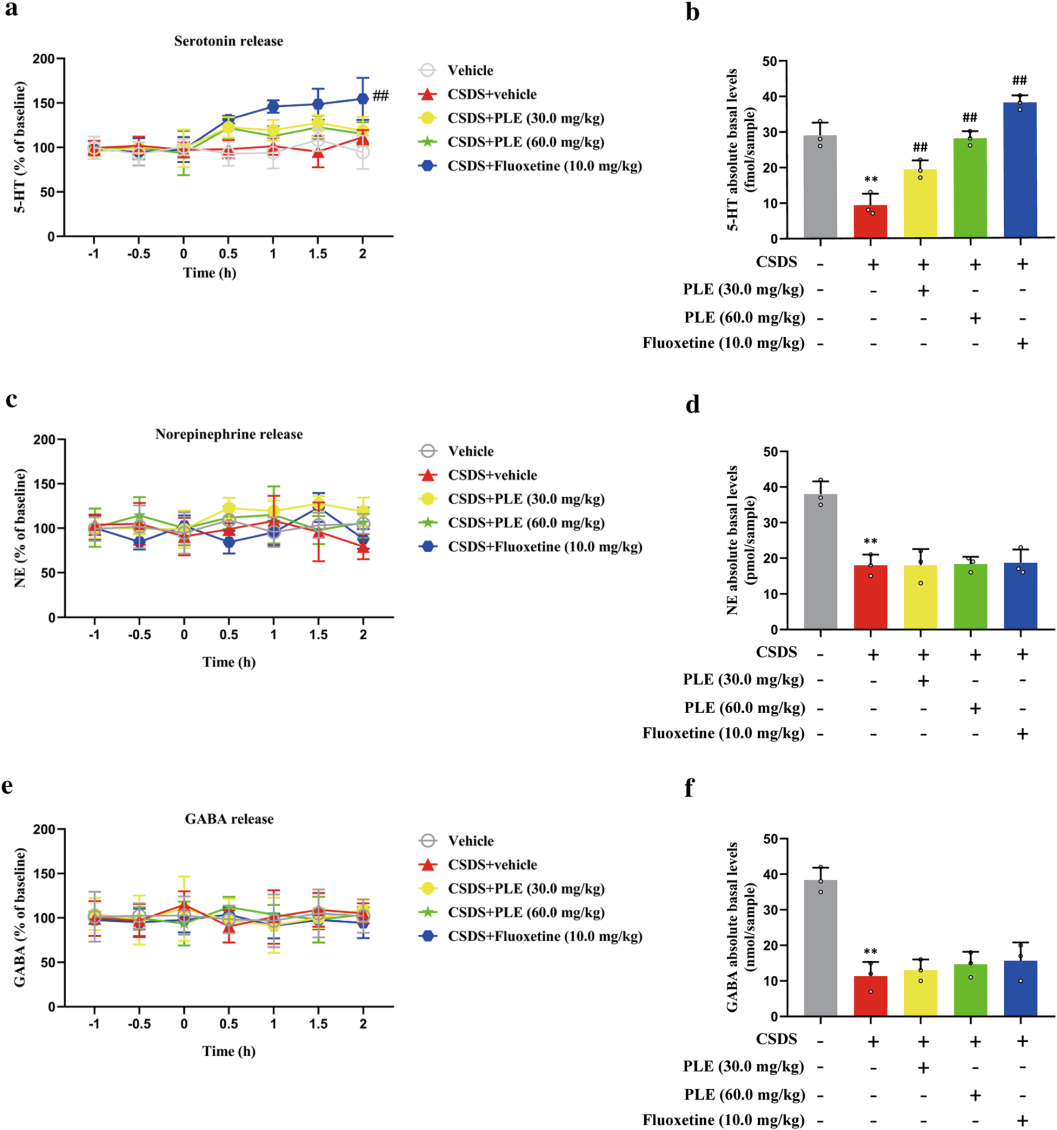
Effects of persimmon leaf extract (PLE) on cortical levels of extracellular neurotransmitters in mice subjected to chronic social defeat stress (CSDS). **a** Levels of the extracellular serotonin receptor (5-HT). **b** Absolute basal levels of 5-HT. **c** Levels of the extracellular norepinephrine (NE). **d** Absolute basal levels of NE. **e** Levels of extracellular gamma-aminobutyric acid (GABA). **f** Absolute basal levels of GABA. Data are presented as mean ± SD (n = 3 in each group). **P < 0.01 vs. Vehicle group; ^##^P < 0.01 vs. CSDS + Vehicle group

We quantified the level of neurotransmitters in the brain of CSDS-exposed mice to see determine how PLE affects extracellular neurotransmitter levels in the cortex. Our results showed that prolonged PLE treatment had no influence on extracellular 5-HT (P > 0.05, Fig. 6a), NE (P > 0.05, Fig. 6c), or GABA (P > 0.05, Fig. 6e) levels, while fluoxetine significantly increased extracellular 5-HT levels (P < 0.01, Fig. 6a). We also observed that CSDS treatment induced a significant decrease in the absolute baseline 5-HT levels compared to mice in the Vehicle group (P < 0.01; Fig. 6b), which were restored by treatment with PLE or fluoxetine (P < 0.01; Fig. 6b). Meanwhile, these treatments did not significantly alter the absolute baseline levels of NE or GABA in the cortex of mice (P > 0.05, Fig. 6d, f). Therefore, PLE only inhibited 5-HT reuptake in the cortex of CSDS-exposed mice.

### PLE increased cortical cAMP, phosphorylated CREB, and BDNF levels in mice exposed to CSDS

**Fig. 7:**
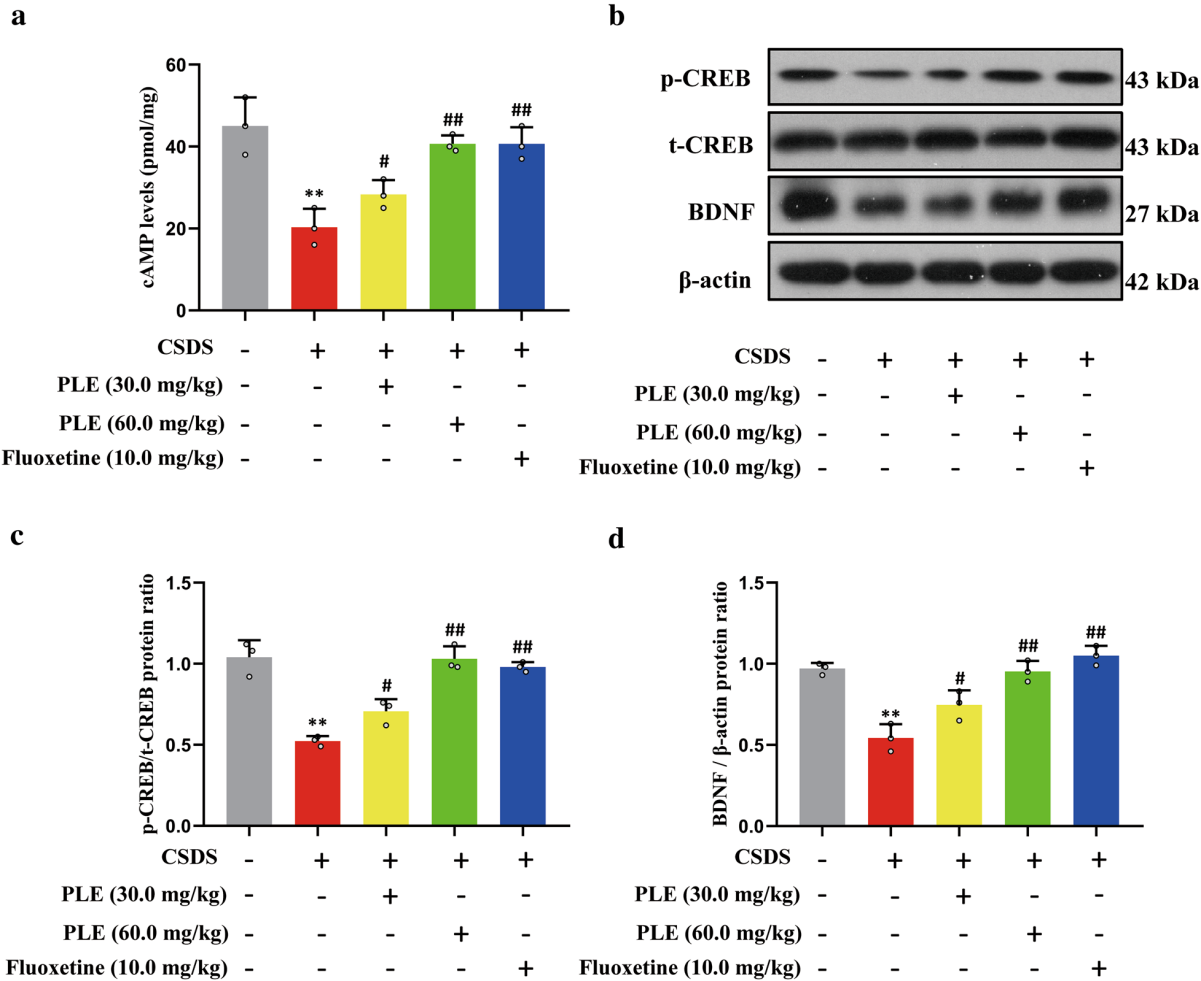
Effects of Persimmon leaf extract (PLE) on cortical levels of cortical cyclic adenosine monophosphate (cAMP), phosphorylated cAMP response element binding protein (p-CREB) and brain-derived neurotrophic factor (BDNF) in mice exposed to chronic social defeat stress (CSDS). **a** cAMP levels. **b** Examples of original Western blot bands showing cortical expression of p-CREB and BDNF. **c** Relative levels of p-CREB. **d** Relative levels of BDNF. Data are presented as mean ± SD (n = 3 in each group). **P < 0.01 vs. Vehicle group; ^#^P < 0.05, ^##^P < 0.01 vs. CSDS + Vehicle group

To further explore the underlying mechanisms, we evaluated cAMP levels in the brain of mice subjected to CSDS. PLE (30 and 60 mg/kg) dramatically increased cortical cAMP levels (P < 0.01, Fig. 7a). Therefore, we investigated the amount of phosphorylated CREB in mice exposed to CSDS. We discovered that CSDS inhibited phosphorylated CREB compared to mice treated with vehicle alone, whereas PLE and fluoxetine restored the loss in CREB phosphorylation without affecting overall CREB level (P < 0.01, Fig. 7b, c). BDNF is a downstream target of CREB, and BDNF deficiency is associated with the pathophysiology of severe depression. In our model, mice treated with vehicle showed high expression of BDNF, while CSDS treatment markedly reduced the expression of BDNF. Furthermore, PLE or fluoxetine treatment significantly restored the loss of BDNF expression (P < 0.05 and P < 0.01, Fig. 7b, d). These results suggested that PLE exerts its antidepressant-like effect through the regulation of the cAMP/CREB/BDNF signaling pathway.

### PLE promoted neurogenesis in mice exposed to CSDS

**Fig. 8:**
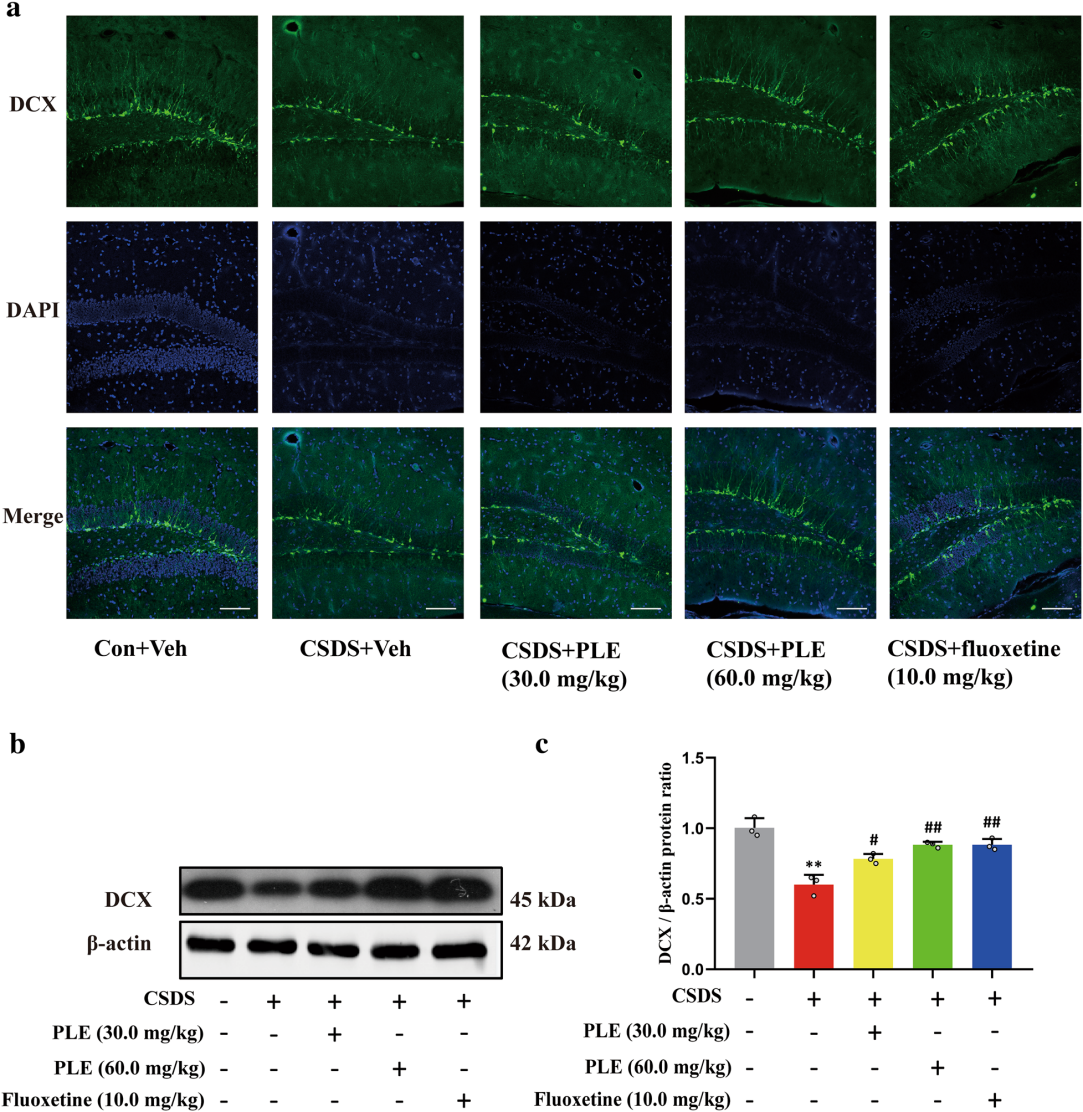
Effect of PLE on cortical doublecortin (DCX) in mice subjected to chronic social defeat stress (CSDS). **a** DCX (green) and DAPI (blue) in the cortex of mice treated with Persimmon leaf extract (PLE) or fluoxetine for 10 days (scale bar: 100 μm). **b** Examples of original western blot bands showing cortical DCX expression levels. **c** Relative levels of DCX. Data are presented as mean ± SD (n = 3 in each group). **P < 0.01 vs. Vehicle group; ^#^P < 0.05, ^##^P < 0.01 vs. CSDS + Vehicle group

PLE activated the cAMP signaling pathway and increased synaptic-related protein expression levels. To investigate whether PLE promotes neurogenesis in the hippocampus of CSDS-subjected mice, we counted the number of DCX^+^ cells in the dentate gyrus using immunofluorescence and quantified DCX expression using western blot. CSDS treatment induced a significant decrease in DCX^+^ cells and neural progenitor cells in the dentate gyrus compared to mice in the Vehicle group, while PLE or fluoxetine treatment restored the loss of DCX^+^ cells and neural progenitor cells in mice (Fig. 8a). Furthermore, CSDS treatment markedly reduced DCX expression in mice, which was reversed by PLE or fluoxetine treatment in the hippocampal cells of mice (P < 0.05 and P < 0.01, Fig. 8b, c) according to western blot findings.

### CSDS led to depression-like behavior and gut microbial changes in mice

**Fig. 9:**
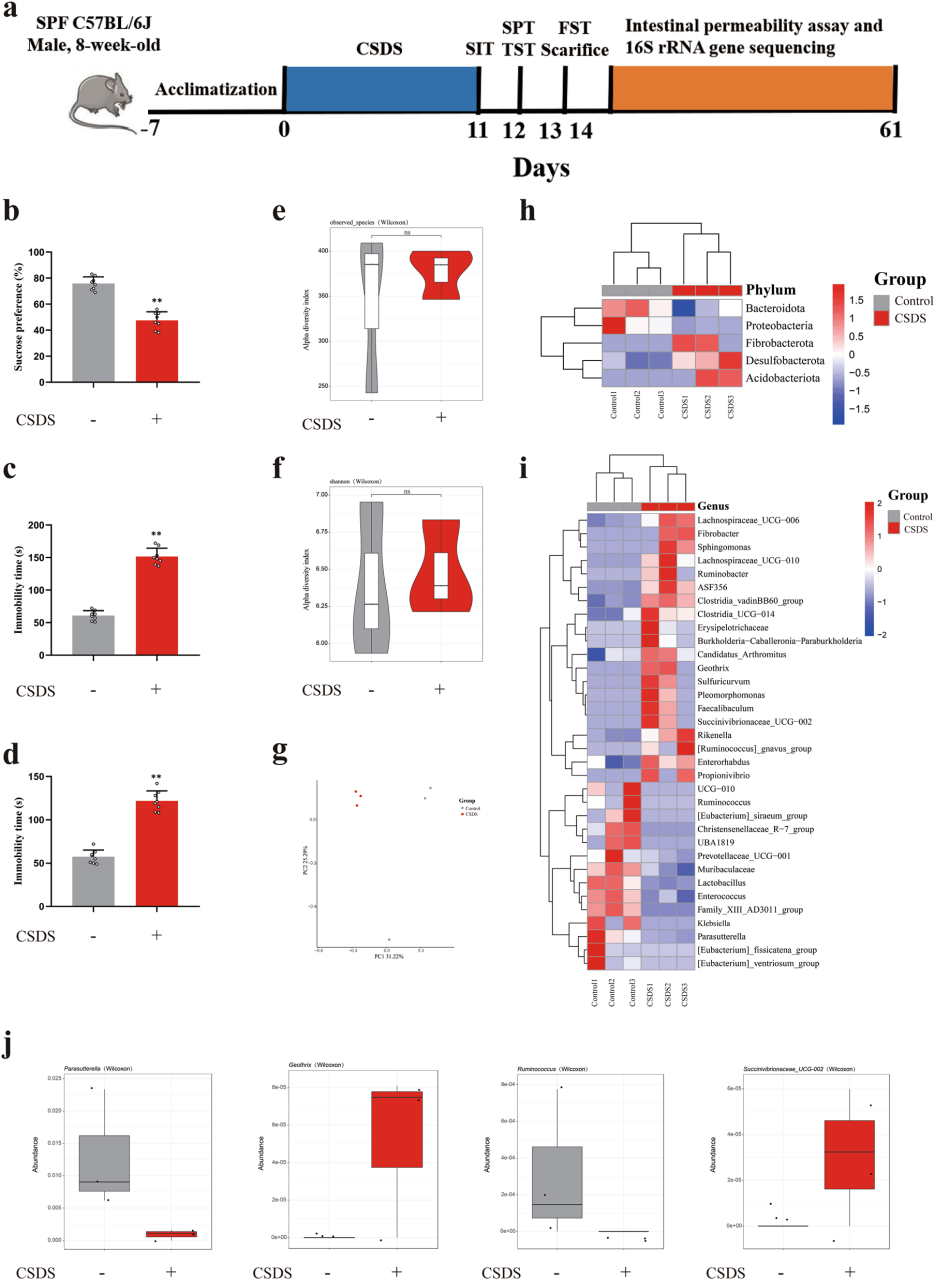
16S rRNA gene sequencing of the gut microbiota in mice. **a** Timeline of the experiments. **b** The sucrose preference ratio in sucrose preference test (SPT). **c** The immobility time in tail suspension test (TST). **d** The immobility time in forced swimming test (FST). **e** Observed species index. **f** Shannon index. **g** PCoA analysis. **h** Heatmap showing the relative abundance of differential flora at the phylum level. Relative abundance of different bacteria at the phylum level. **i** Heatmap showing the relative abundance of *Parasutterella*, *Ruminococcus*, *Geothrix* and *Succinivibionaceae_UCG-002* at the genus level. Data are presented as mean ± SD (n = 3 in each group). *P < 0.05 vs. Control group

The occurrence and development of depression was accompanied with a change in the composition of the gut microbiota. Therefore, to further study the relationship between gut microbial symbiosis and anti-depression effect of PLE, we collected fecal samples and identified the fecal microbial communities of C57BL/6J mice and CSDS-treated mice by 16S rRNA gene sequencing. CSDS-subjected mice showed anhedonia-like behavior in the SPT, and despair-like behavior in the TST and FST (Fig. 9b–d). There were no differences in the total number of microbial species or diversity between the two groups (P > 0.05, Fig. 9e, f, h, i). CSDS mice presented a distinct fecal microbiota distribution which differed from that of the control C57BL/6J mice (Fig. 9g). The differences between the two groups were caused by a lower abundance of *Parasutterella* and *Ruminococcus* at the genus level in the fecal microbiota of CSDS-subjected mice, while *Geothrix* and *Succinivibionaceae_UCG-002* were increased (Fig. 9j).

### PLE alleviated depressive behavior by modulating intestinal microbes in mice exposed to CSDS

**Fig. 10:**
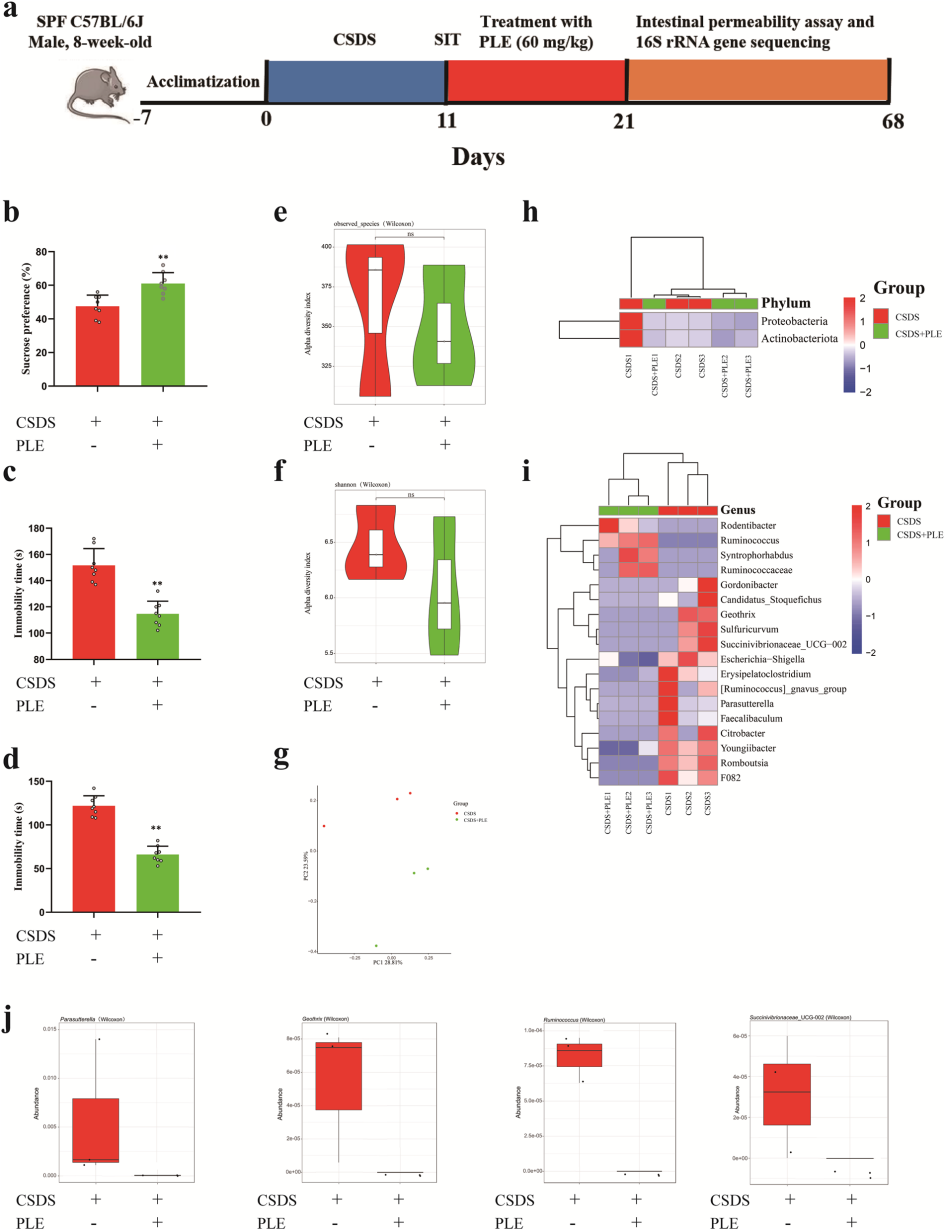
PLE alleviates depressive behavior by modulating intestinal microbes in mice exposed to CSDS. **a** Timeline of the experiments. **b** The sucrose preference ratio in sucrose preference test (SPT). **c** The immobility time in tail suspension test (TST). **d** The immobility time in forced swimming test (FST). **e** Observed species index. **f** Shannon index. **g** PCoA analysis. **h** Heatmap showing the relative abundance of differential flora at the phylum level. Relative abundance of different bacteria at the phylum level. **i** Heatmap showing the relative abundance of *Parasutterella*, *Ruminococcus*, *Geothrix* and *Succinivibionaceae_UCG-002* at the genus level. Data are presented as mean ± SD (n = 3 or 8 in each group). *P < 0.05 vs. Control group

The therapeutic effect of traditional antidepressants may influence the gut microbiota [40]. We gavaged CSDS-subjected mice with PLE and evaluated the effects of PLE on gut microbial dysbiosis. Following gavage with PLE, the depression-like behavior was rescued (Fig. 10b–d) and there were no differences between the two groups in the total number of microbial species or in species diversity (P > 0.05, Fig. 10e, f, h, i). PLE-treated mice also represented a distinct fecal microbiota cluster, which differed from that of CSDS-subjected mice not receiving PLE (Fig. 10 g). At the genus level, *Parasutterella*, *Ruminococcus*, *Geothrix*, and *Succinivibionaceae_UCG-002* in the PLE-treated mice fecal microbiota were decreased (Fig. 10j). Thus, we speculated that *Geothrix* and *Succinivibionaceae_UCG-002* contributed to the anti-depressant effect of PLE.

## Discussion

**Fig. 11:**
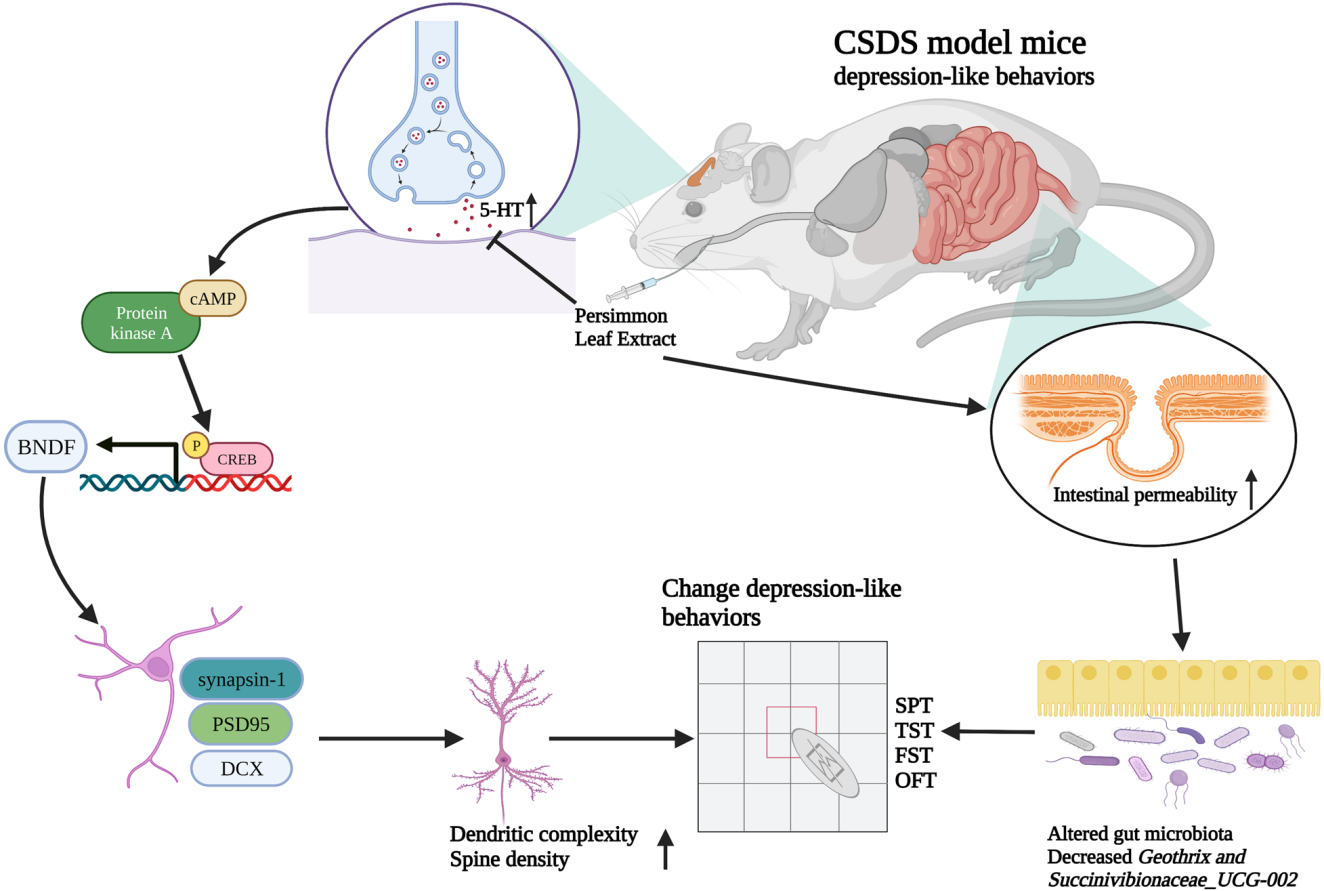
PLE alleviated CSDS-induced depressive behaviors by inhibiting serotonin reuptake and influenced the composition of the fecal microbiota

PLE exerted antidepressant-like effects in CSDS-subjected mice by inhibiting 5-HT reuptake as supported by the following findings: (1) PLE restored sucrose preference in the SPT and shortened the duration of immobility in both the TST and FST but did not affect locomotor activity in the OFT. (2) PLE significantly enhanced dendritic length, branching points, and dendritic spine density in the cortex. (3) PLE significantly increased the expression of PSD95 and synapsin-1 in the cortex of mice subjected to CSDS. (4) PLE inhibited 5-HT reuptake in CSDS-subjected mice. (5) PLE increased the level of cAMP and BDNF expression, as well as activated CREB phosphorylation in the cortex of CSDS-subjected mice. (6) PLE increased the number of DCX^+^ cells in the dentate gyrus, facilitating neurogenesis in mice subjected to CSDS. (7) PLE could reverse the change of microbial species in CSDS-exposed mice. Thus, the present study provided evidence that PLE exerted its antidepressant-like activity in mice subjected on CSDS by inhibiting 5-HT reuptake, regulating the cAMP/CREB/BDNF signaling pathway, and facilitating neurogenesis. The antidepressant-like effect of PLE may be related to affecting the gut microbiota of CSDS-exposed mice. A summary of the anti-depression of PLE in CSDS-exposed mice and potentially related signaling pathways are shown in Fig. 11.

During life activities, people constantly interact with each other, social challenges appear to be one of the most common stresses in humans and social animals. Mice repeatedly exposed to social defeat stress develop anhedonia, behavioral despair, and social avoidance. CSDS is a widely recognized animal model that simulates the etiology of human depression, it has been extensively utilized to investigate the pathogenesis of depression [41]. In our study, mice exposed to CSDS displayed depressive-like behaviors, including social avoidance, anhedonia, and extended immobility in the TST and FST.

Flavonoids have potential as treatments for emotional disorders such as anxiety and depression, for example, they may improve depression in young people [42, 43]. In addition, extensive research suggests that flavonoids are the most significant therapeutic components in Persimmon leaves [44, 45]. Based on these data, we hypothesized that Persimmon leaves exert antidepressant properties. Not unexpectedly, PLE administration significantly reduced behavior deficits in CSDS-subjected animals in the SPT, TST, and FST without affecting locomotor activity. These findings validated the antidepressant-like action of PLE in mice. Thus, this study is the first to show that PLE alleviated depressive-like behavior in mice. However, while our study supports the possible applicability of PLE, further testing, particularly rigorous toxicological studies of PLE, must be conducted in the future.

5-HT, the most important neurotransmitter in the onset and progression of depression, may regulate and govern a range of signaling pathways in the nervous system [23, 46, 47]. However, other neurotransmitters, such as dopamine and glutamate, are linked to Alzheimer’s and Parkinson’s disease, but not to depression [48]. 5-HT signaling and 5-HT receptors tightly regulate neurotrophic factors levels and adult neurogenesis. The cAMP/CREB/BDNF signaling network is a canonical pathway responsible for the physiological effects of 5-HT [49, 50]. It is a common target mechanism for many classes of antidepressant [51]. Previous studies showed that the levels of 5-HT could increase the expression of BDNF in the brain of rat, and BDNF also affects serotonergic neurotransmission in both baseline and activated situations [52]. In our study, we discovered that prolonged PLE treatment had no influence on extracellular 5-HT levels, but could increase the absolute baseline 5-HT level in cortex of CSDS-exposed mice, which suggested that PLE only inhibited 5-HT reuptake in the cortex. Therefore, PLE may exert its antidepressant-like effect by inhibiting 5-HT reuptake and regulating the cAMP/CREB/BDNF signaling pathway in cortex of CSDS-exposed mice. Inhibiting the cAMP/CREB/BDNF pathway or the elimination of critical cAMP/CREB/BDNF proteins could confirm the antidepressant-like action mechanism of PLE. It has been shown that cortex is a node in emotion regulation in several studies, activation of the cAMP/CREB/BDNF signaling pathway in the cortex is critical in antidepressant, but the hippocampus is more related with memory, thus we focus on the cortex.

BDNF is a powerful neurotrophin that helps neurons retain their shape and regulates brain plasticity. It is also important to convert synaptic activity into long-term synaptic memory [53]. The pathological process of depression is related to a lack of synaptic plasticity [54]. Depression raises blood corticosterone levels, lowers BDNF levels, and inhibits neurogenesis [55]. Antidepressants that enhance BDNF levels and neurogenesis in the cortex and hippocampus include fluoxetine, a selective 5-HT reuptake inhibitor [56]. Inhibition of neurogenesis or neural plasticity reduces the impact of antidepressants, suggesting that neurogenesis and neural plasticity participate in the amelioration of depressive-like behaviors [57]. Numerous studies have revealed that CREB and BDNF are essential regulators of neurogenesis, neural plasticity, and neuronal survival [58]. Meanwhile, our previous study found that a new phosphodiesterase 4 inhibitor, FCPR16, can stimulate the cAMP/CREB/BDNF signaling pathways in mice subjected to chronic unpredictable moderate stress, resulting in an increase in DCX^+^ cells in the hippocampus and improved neurogenesis [59]. Stress reduces the expression of neurotrophic factors (e.g., BDNF) in the limbic and cortical regions, which supports the neurotrophic theory [53]. Researchers have shown a decrease in BDNF in the hippocampus and prefrontal cortex of rodents exposed to stressful situations [60, 61]. Herein, we revealed that PLE raised BDNF levels. Thus, PLE may have neuroprotective benefits by modulating the expression of BDNF in the brain.

The creation and removal of dendritic spines requires proper expression of synaptic proteins [62]. In our previous studies, we discovered that CSDS reduced dendritic length, branching points, and dendritic spine density [63]. Here, treatment with PLE effectively reversed these morphological changes. Furthermore, PLE affected cytoskeleton dynamics and changed dendritic shape by stimulating cAMP/CREB/BDNF signaling pathways. Dendritic spines play a crucial role in neural transmission and neuroplasticity [64]. Changes in dendritic morphology contributed to prolonged stress-induced behavioral abnormalities, while depression decreased spine density and structure [65]. The mechanisms behind the atrophy of dendritic spines and branching in response to chronic stress are currently unknown, but reduction in neurotrophic factor levels and synaptic protein production could contribute to the consequences of stress [66]. BDNF may regulate dendritic length and branching in neurons, while CREB mediates the beneficial effects of BDNF on dendritic length and complexity [67]. We discovered that PLE increased the levels of synapsin-1 and PSD95, which involved in the development, morphology, anti-inflammatory (Additional file 1: Fig. S1) and functions of synapses [68]. Therefore, the increased expression of synaptic proteins is responsible for the morphological changes caused by PLE. Similarly, PLE increased the number of DCX^+^ cells in the hippocampus of mice, which suggests that PLE improves neurogenesis in CSDS-subjected mice.

In CSDS-treated mice, 16S rRNA analysis revealed aberrant composition of the intestinal microbiota. The composition of several microbiota was altered in CSDS-exposed mice models exhibiting depression-like symptoms compared to control C57BL/6J mice in this study. *Geothrix* and *Succinivibionaceae_UCG-002* were considerably reduced in CSDS-treated mice compared to control C57BL/6J animals at the genus level. The gut microbiota of MDD rodent model and healthy controls were shown to be considerably different, with significant changes in the relative abundance of *Geothrix* and *Succinivibionaceae_UCG-002*. We also discovered that treatment with PLE could rescue depressive-like behaviors and lower the abundance of *Geothrix* and *Succinivibionaceae_UCG-002* in CSDS-exposed mice, indicating that *Succinivibionaceae_UCG-002* may play a role in the antidepressant effects of PLE. Despite this, further research is needed to validate the role of *Succinivibionaceae_UCG-002* in the antidepressant activity of PLE.

## Conclusions

Our study showed that PLE exerts an antidepressant-like effect in CSDS-subjected mice and improved neurogenesis. Furthermore, the observed behavioral effects were associated with the inhibition of 5-HT reuptake, the activation of the cAMP/CREB/BDNF signaling pathway, and the upregulation of cortical synapsin-1 and PSD95 expression. Simultaneously, PLE influenced the composition of the fecal microbiota in CSDS-subjected mice. Taken together, our results indicate that PLE has antidepressant-like effects and should be considered as a candidate anti-depressive treatment.

## Supplementary Information

**Additional file 1.** The effects of Persimmon leaf extract (PLE) on inflammatory cytokines in mice after chronic social defeat stress.

## Data Availability

The data used to support the current study are available from the corresponding author on reasonable request.

## Abbreviations

5-HT1A: Serotonin receptor 1 A
BDNF: Brain-derived neurotrophic factor
cAMP: Cyclic adenosine monophosphate
CREB: cAMP response element-binding protein
CSDS: Chronic social defeat stress
DCX: Doublecortin
ELISA: Enzyme-linked immunosorbent assay
FST: Forced swim test
GABA: Gamma-aminobutyric acid
HPLC: High-performance liquid chromatography
NE: Norepinephrine
OFT: Open field test
PFA: Paraformaldehyde
PKA: Protein kinase A
PLE: Persimmon leaf extract
PSD95: Postsynaptic density protein
SPT: Sucrose preference test
TST: Tail suspension test
