# Supplementary material for: Persimmon leaf extract alleviates chronic social defeat stress-induced depressive-like behaviors by preventing dendritic spine loss via inhibition of serotonin reuptake in mice
Source: Chin Med. 2022 Jun 6;17:65. doi: 10.1186/s13020-022-00609-4 (PMC9172164; doi:10.1186/s13020-022-00609-4)
Supplement: Supplementary file 1 — Additional file 1. The effects of Persimmon leaf extract (PLE) on inflammatory cytokines in mice after chronic social defeat stress. [file 13020_2022_609_MOESM1_ESM.docx]

**Persimmon leaf extract alleviates chronic social defeat stress-induced depressive-like behaviors by preventing dendritic spine loss via inhibition of serotonin reuptake in mice**

Hui Yu^1†^, Shumin Shao^2†^, Junnan Xu^3†^, Haibiao Guo^4^, Zhangfeng Zhong^2*^, Jiangping Xu^1*^

^1^Provincial Key Laboratory of New Drug Screening, School of Pharmaceutical Sciences, Southern Medical University, Guangzhou, 510515, China

^2^Macau Centre for Research and Development in Chinese Medicine, Institute of Chinese Medical Sciences, University of Macau, Macao SAR 999078, China

^3^Department of Neurobiology, School of Basic Medical Sciences, Southern Medical University, Guangzhou, 510515, China

^4^Hutchison Whampoa Guangzhou Baiyunshan Chinese Medicine Co., Ltd., Guangzhou 510515, China

†Hui Yu, Shumin Shao, and Junnan Xu contributed equally to this work

Correspondence: [jpx@smu.edu.cn;](mailto:jpx@smu.edu.cn;) [zhangfengzhong@um.edu.mo](mailto:zhangfengzhong@um.edu.mo)

**Materials**

IL-6 ELISA kit (EK0411, BOSTER, China), IL-10 ELISA kit (EK0417, BOSTER, China), TNF-α ELISA kit (EK0527, BOSTER, China) and IL-1β ELISA kit (EK0394, BOSTER, China).

**Methods**

**Enzyme-linked immunosorbent assay (ELISA)**

The cerebral cortex of mice was quickly dissected on ice, homogenized and centrifuged at 12000×rpm for 15 min at 4ºC. Supernatants were used to determine the concentrations of IL-6, IL-10, TNF-α and IL-1β by ELISA kits according to the product protocols. Data were all normalized to total protein.


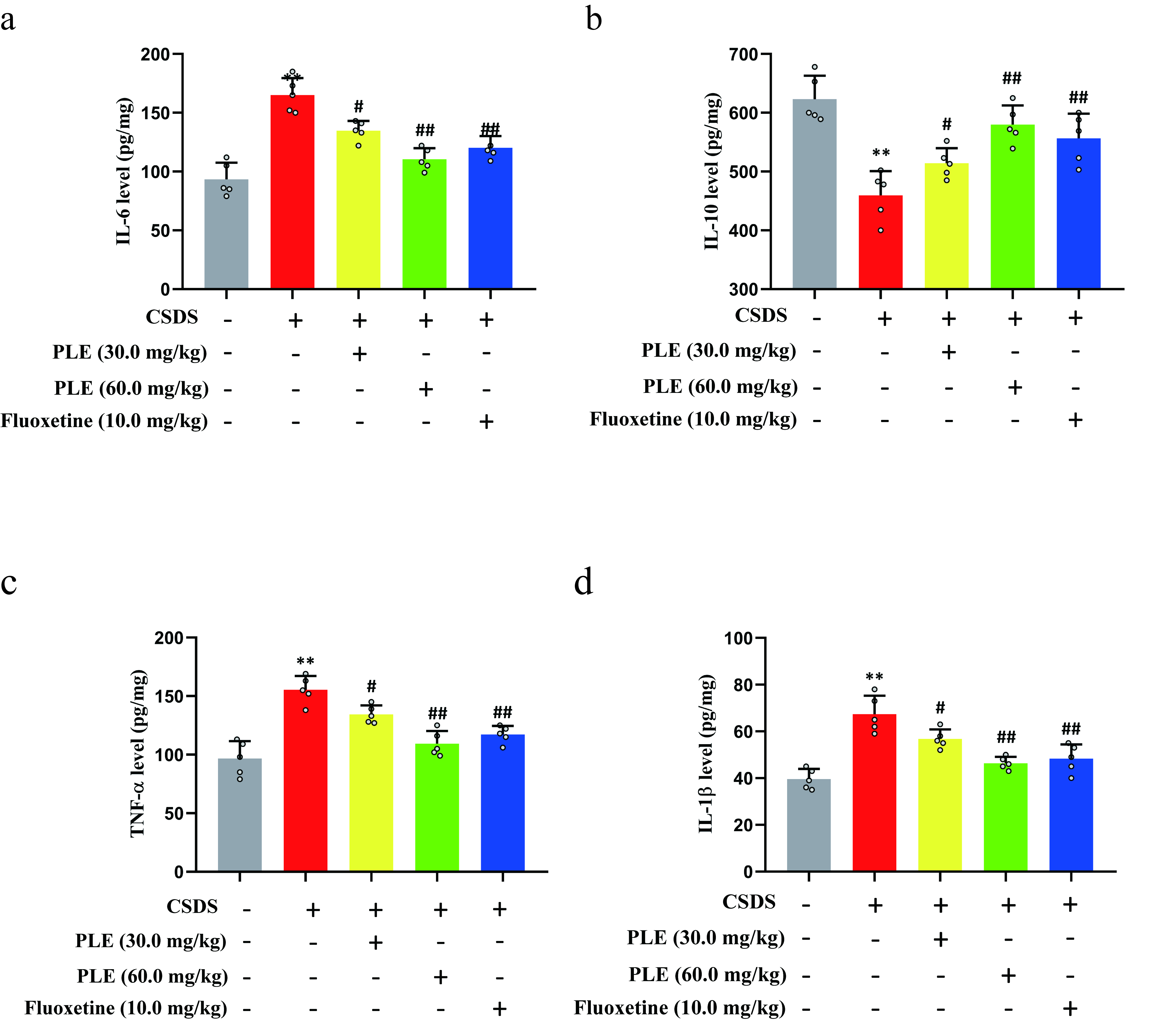


Fig. S1. Effect of Persimmon leaf extract (PLE) on inflammatory cytokines in chronic social defeat stress (CSDS)-subjected mice in cortex. a: The level of IL-6. b: The level of IL-1β. c: The level of TNF-α. d: The level of IL-10. Data are expressed as Mean ± SD (n=5). ^**^*p*<0.01 versus control group, ^#^*p*<0.05 and ^##^*p*<0.01 versus CSDS group.
